# The role of *Weizmannia (Bacillus) coagulans* LMG S-31876 in treating IBS-diarrhea

**DOI:** 10.3389/fnut.2023.1310462

**Published:** 2024-02-05

**Authors:** Ranjith Kumar Kallur, Sreenadh Madapati, Ankita Mathur, Sourish Bhattacharya

**Affiliations:** ^1^Abode Biotec India Private Ltd., Hyderabad, India; ^2^Process Design and Engineering Cell, CSIR-Central Salt and Marine Chemicals Research Institute, Bhavnagar, India

**Keywords:** *Weizmannia (Bacillus) coagulans* LMG S-31876, probiotics, irritable bowel syndrome, diarrhea, clinical trials

## Abstract

**Introduction:**

Irritable bowel syndrome (IBS) is a common gastrointestinal condition. Some studies have shown the efficacy of probiotics in the treatment of irritable bowel syndrome (IBS). *Weizmannia (Bacillus) coagulans* LMG S-31876 has been marketed as a dietary ingredient, but to date, its efficacy in diarrhea-predominant irritable bowel syndrome (IBS) condition has not been clinically elucidated. Thus, a double-blind placebo-controlled multi-centered trial was planned to evaluate the safety and efficacy of *Weizmannia (Bacillus) coagulans* LMG S-31876 in diarrhea-predominant IBS patients.

**Experimental design:**

Study participants (*n* = 50) diagnosed with IBS prominent symptoms that include abdominal pain and other GI-related symptoms were treated with ProBC Plus (2 billion CFU) along with a placebo capsule once daily for approximately 8 weeks. Study participants were evaluated for the treatment success determined by the differences in stool consistency and frequency per day between the intervention and placebo groups over the study period.

**Results:**

The vital signs and the biochemistry parameters were under the normal range; the other parameters showed a significant result as compared to the placebo during the study period.

**Conclusion:**

This study depicts a significant decline in the clinical symptoms such as abdominal pain, bloating, diarrhea, and frequency of the stool as compared to the placebo. All the parameters such as hematology, lipid profile, and vital signs were in the normal range during the supplementation of ProBC Plus for a period of 8 weeks. Furthermore, the study verified that *Weizmannia (Bacillus) coagulans* LMG S-31876 and its probiotic product ProBC Plus at a dose of 2 billion/CFU/day has a prominent action in the relief from the clinical symptoms of IBS-D. Therefore, the product is intended safe to utilize for IBS-related symptoms.

**Clinical trial registration**: The clinical study has been registered with CTRI/2023/01/048644 with https://ctri.nic.in/Clinicaltrials/pmaindet2.php?trialid=77708&EncHid=24313.96864&userName=CTRI/2023/01/048644 [CTRI/2023/01/048644].

## 1 Introduction

IBS (irritable bowel syndrome) with predominant diarrhea (IBS-D) is a frequent gastrointestinal disorder that crucially impairs the quality of life for the affected individuals. These are characterized by abdominal pain, changes in bowel habits, and frequent diarrhea. IBS-D represents a multiplex challenge for the patients seeking relief and for the clinicians who strive to provide treatment options. In recent years, the utilization of probiotics has been emerging to alleviate the symptoms and improve the wellbeing of individuals suffering from IBS. IBS (irritable bowel syndrome) is a common and chronic gastrointestinal (GI) disorder that constitutes the largest group in gastroenterology (1). The symptoms can be stimulated by changes in GI functions caused by bacterial infections, an altered diet, or stress. Subsequently, it also impacts immune activation and inflammation. The major symptoms of the disorder manifest as bloating, reflux, constipation, vomiting, diarrhea, abdominal pain, and cramping (2). Diarrhea is one of the major problems defined as the commotion of the water content and deviation from the normal functioning of the physiologic processes. It is categorized as acute, persistent, and chronic (3). Diarrheal disease is the second leading cause of death in children under the age of 5 and has also become a major gastrointestinal problem in adults in recent times (4).

This disease exhibits altered gut flora that may lead to disordered GI function. The fecal flora of IBS patients differs from that of normal patients. Some individuals with IBS may harbor bacterial overgrowth, and their symptoms may be ameliorated by its eradication. GI disorders are not life-threatening, but they do impact general contentment and quality of life (QOL) (5). Clinical intervention typically includes patient education, reassurance, and dietary modification to alleviate symptoms (1), all of which can be unsatisfactory for patients who are facing this lifelong disease.

There are many readily available solutions for the treatment of this problem, which include the utilization of ORS (oral rehydration solutions), antibiotics, and gut-suppressing agents. Probiotics are live microorganisms that maintain immunologic equilibrium and impart health benefits to the host. The major mechanisms include the exclusion of pathogenic microbes (competitively), a hindrance to the pathogenic microbes and antimicrobial substance production, and also modulation of the immune system (6).

Various species of bacteria are categorized into lactic acid producers, i.e., *Lactobacillus, Bifidobacterium, Peptostreptococcus, Akkermansia,* and *Bacillus coagulans*. To maintain the conventional microflora within the gut, these species are important to retain (7). *Bacillus (Weizmannia) coagulans* are naturally distributed in various fermented foods. These strains have been established as major clinical symptoms, such as IBS and other disturbances in bowel movements, and have also improved the microbiome.

The prevalence of IBS varies by geographic region but has been reported in 1–18% of the general population (4, 8). The British Society of Gastroenterology published their 2021 guidelines for the treatment of IBS and recommended probiotics, in general, as a first-line treatment, but as their analysis pooled dissimilar strains of probiotics, they could not identify which specific probiotics might be useful (9). Choosing an appropriate probiotic for patients can be challenging due to the diversity of different types of available probiotics, efficacy is strain-specific and the paucity of randomized clinical trials for some probiotic strains (10, 11). Unfortunately, many meta-analyses have not followed these recommendations and have not been able to determine which specific probiotic strains might be effective for IBS patients (12–15).

Kumar et al. (16) reported the potential of probiotics in alleviating symptoms and improving overall gut health. Specifically, these studies underscore the importance of understanding the intricate interplay between the microbial community and the symptomatic manifestations of IBS (16). Dale et al. (17) reported the role of probiotic interventions in ameliorating specific symptoms commonly associated with IBS, contributing to the growing body of evidence supporting probiotics as a viable therapeutic option. Wang et al. (18) conducted a randomized controlled trial investigating the efficacy of probiotics in alleviating diarrhea and improving stool frequency in IBS patients. Barraza-Ortiz et al. (19) delve into the intricate relationship between probiotics and gut microbiota composition, providing insights into the mechanisms through which probiotics may exert their beneficial effects. This understanding is crucial for unraveling the complex dynamics that influence the efficacy of probiotic interventions in managing IBS (19). Furthermore, Selvaraj et al. (20) reported the long-term effects of probiotic supplementation on the quality of life in IBS patients. Their findings suggest sustained improvements, highlighting the potential of probiotics not only in symptom relief but also in enhancing overall wellbeing for individuals grappling with the challenges of IBS (20).

Among the various strains of probiotics, *Weizmannia (Bacillus) coagulans* LMG S-31876 has gained attention because of its ability to survive in harsh gastric conditions due to its ability to form spores and to provide protection to the host. *Weizmannia (Bacillus) coagulans* LMG S-31876 is well characterized; it is a member of the subspecies *Bacillus* species and is a spore-forming bacterium isolated from fermented rice samples. The strain is safely deposited with the reference code ProBC Plus. The accession numbers obtained from the Belgian Coordinated Collections of Microorganisms/Laboratorium voor Microbiologie (BCCM/LMG) and Microbial Type Culture Collection-International Depositary Authority (MTCC-IDA) under the Budapest Treaty are LMG S-31876 and MTCC 25396, respectively. It is also deposited with the American Type Culture Collection (ATCC); the assigned number is ATCC SD-7789 (21). In addition to these preliminary findings, evaluation of the efficacy and safety of *Weizmannia (Bacillus) coagulans* LMG S-31876-based interference remains indispensable.

To address this gap, this clinical trial study evaluates the effect of ProBC Plus in patients diagnosed with IBS-D and, subsequently, provides robust and substantial information in the management of IBS-D, thereby enhancing the quality of life for the affected individuals. Gut microbiota plays a crucial role in gastrointestinal health, and its disruption leads to imbalances in IBS-D.

## 2 Materials and methods

The study was conducted at Medstar Speciality Hospital, Bengaluru, Karnataka.

### 2.1 Study design and Its criteria

**Figure 1:**
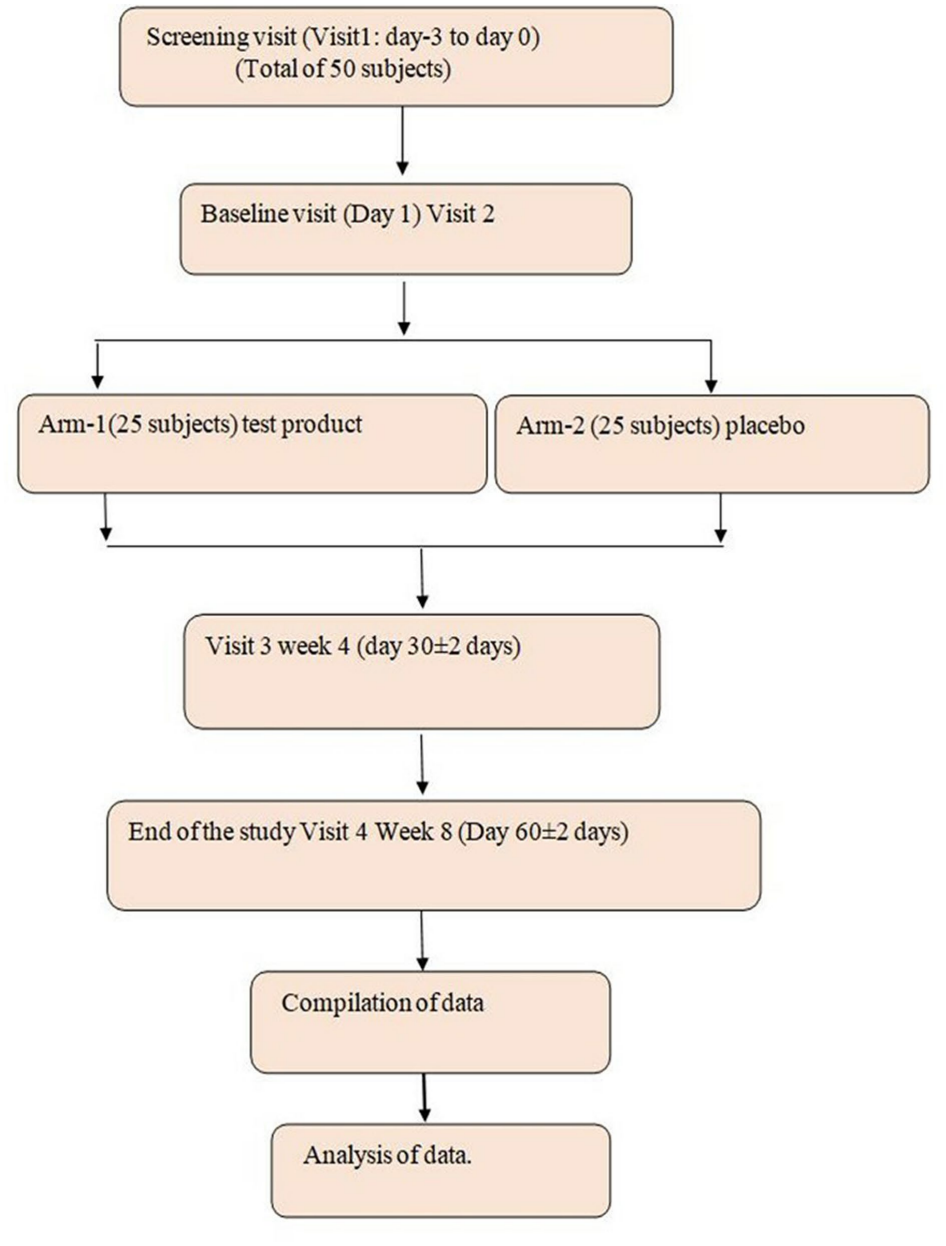
Flow chart of the study procedure.

This study is a randomized, double-blind, placebo-controlled parallel group to evaluate the efficacy and safety of the investigational product (IP) *B. coagulans* LMG S-31876 (10^6^–10^8^ billion CFU) in the treatment of IBS. The study duration for each subject is up to 8 weeks. A total of 50 study participants will be included in the study. This consisted of a total of 4 visits including screening visits: visit 1: screening visit: (−5 to −2 days); visit 2: baseline (Day 1); visit 3: week 4; and visit 4: week 8 (Figure 1).

#### 2.1.1 Inclusion criteria

The inclusion criteria were as follows: The study participants should be (i) either male or female; (ii) aged between 18 and 65 years; (iii) signed an informed consent; (iv) IBS-Diarrhea (IBS-D) patients satisfying Rome III criteria. Symptoms such as recurrent abdominal pain or discomfort, 3 days per month in the last 3 months (12 weeks). The criteria are fulfilled with symptoms onset 6 months before diagnosis—improvement with defecation, onset associated with a change in stool frequency, and onset associated with a change in stool form (appearance).

#### 2.1.2 Exclusion criteria

The study participants were excluded from the study if they were on antibiotics, utilization of complementary and alternative medicines for IBS symptoms, and pregnant or lactating women. Study participants will be excluded if the treatment limits of permitted medication are more than 2 days/week during the study period, requiring treatments with non-permitted medication. IBS study participants with untreated lactose intolerance, regular utilization of probiotics, study participants with rectal bleeding, and allergies to active ingredients are excluded from the study.

#### 2.1.3 Ethical considerations

The study is designed, conducted, analyzed, and reported by regulatory and ethical guidelines (ICH GCP, Indian GCP, and Schedule-Y). The clinical study is registered in CTRI (Clinical Trial Registry India) with CTRI number CTRI/2023/01/048644.

### 2.2 Investigational product

There were two groups for the study. Group 1: Probiotic containing *B. coagulans* LMG S-31876 strain (ProBC Plus) (10^6^–10^8^ CFU), 2 billion CFU/Capsule/day; Group II: Placebo.

### 2.3 Method of analysis and formulation

All the study participants were treated with 2 billion CFU/Capsule/day to be re-constituted in water to be consumed in the morning and evening (one each).

#### 2.3.1 Study tools

The study consisted of an assessment of Abdominal pain VAS (0–10 scale), IBS Symptom Severity Scale (IBS-SSS), Bristol Stool Form Scale (BSFS), stool frequency, assessment of blood hematological parameters, and lipid profile and vital signs were analyzed.

#### 2.3.2 Efficacy and safety assessment

The primary outcomes include the difference in the overall symptom relief between the intervention group and placebo group as assessed by IBS-SSS (time frame: 8 weeks). Secondary outcomes include the measurement of (a) differences in stool consistency and frequency/day between the intervention group and the placebo group over the study period and (b) differences in hematological parameters and lipid profile between the intervention and placebo groups over the study period (time frame: 8 weeks).

#### 2.3.3 Statistical significance

The baseline values for VAS, Bristol Stool Form, hematology, and biochemical parameters were compared to the treatment groups by appropriate statistical tools (SPSS).

The statistical data obtained for the study were analyzed utilizing two sample *T*-tests for the comparative analysis of the results between the baseline and the end of the treatment. *p*-values are based on the analysis of the covariance (ANCOVA) model with the change from the baseline values. *p*-values (drug vs. placebo) at week 8 are based on the ANCOVA model.

## 3 Results

A total of 50 study participants were enrolled in the study. The mean age was between 18 and 65, and study participants were treated with ProBC plus. There was no medical history of the study participants utilized for the study. The study participants were divided equally into placebo and treatment groups (Supplementary Table S1).

### 3.1 Vital signs and physical examination

**Figure 2:**
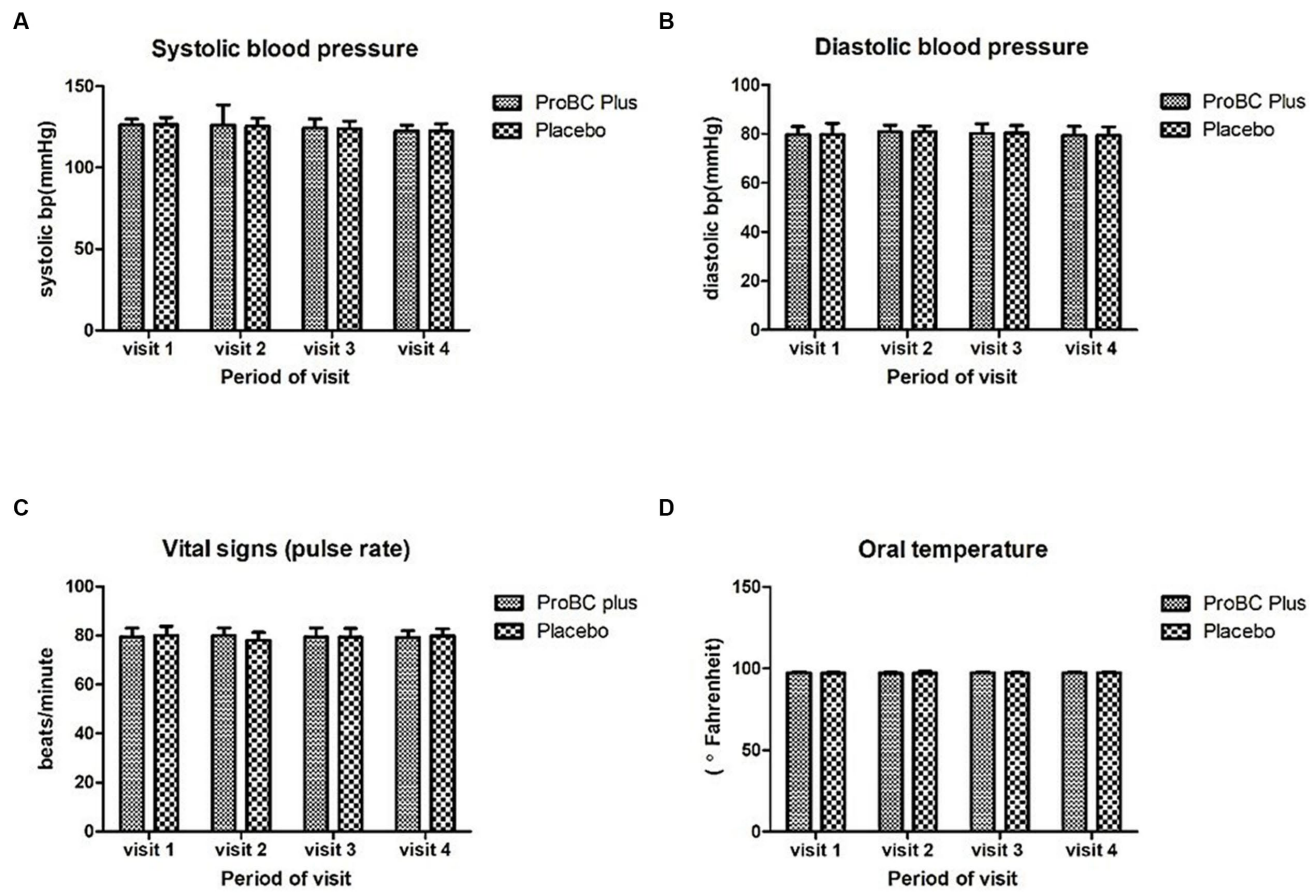
Measurements of vital signs: **(A)** systolic blood pressure, **(B)** diastolic blood pressure, **(C)** pulse rate, and **(D)** oral temperature.

Vital signs such as oral temperature, blood pressure, and pulse rate were found to be statistically significant from the baseline. Figure 2 represents the vital signs for the treatment and placebo groups.

### 3.2 Evaluation of laboratory parameters

#### 3.2.1 Analysis of hematology parameters

**Figure 3:**
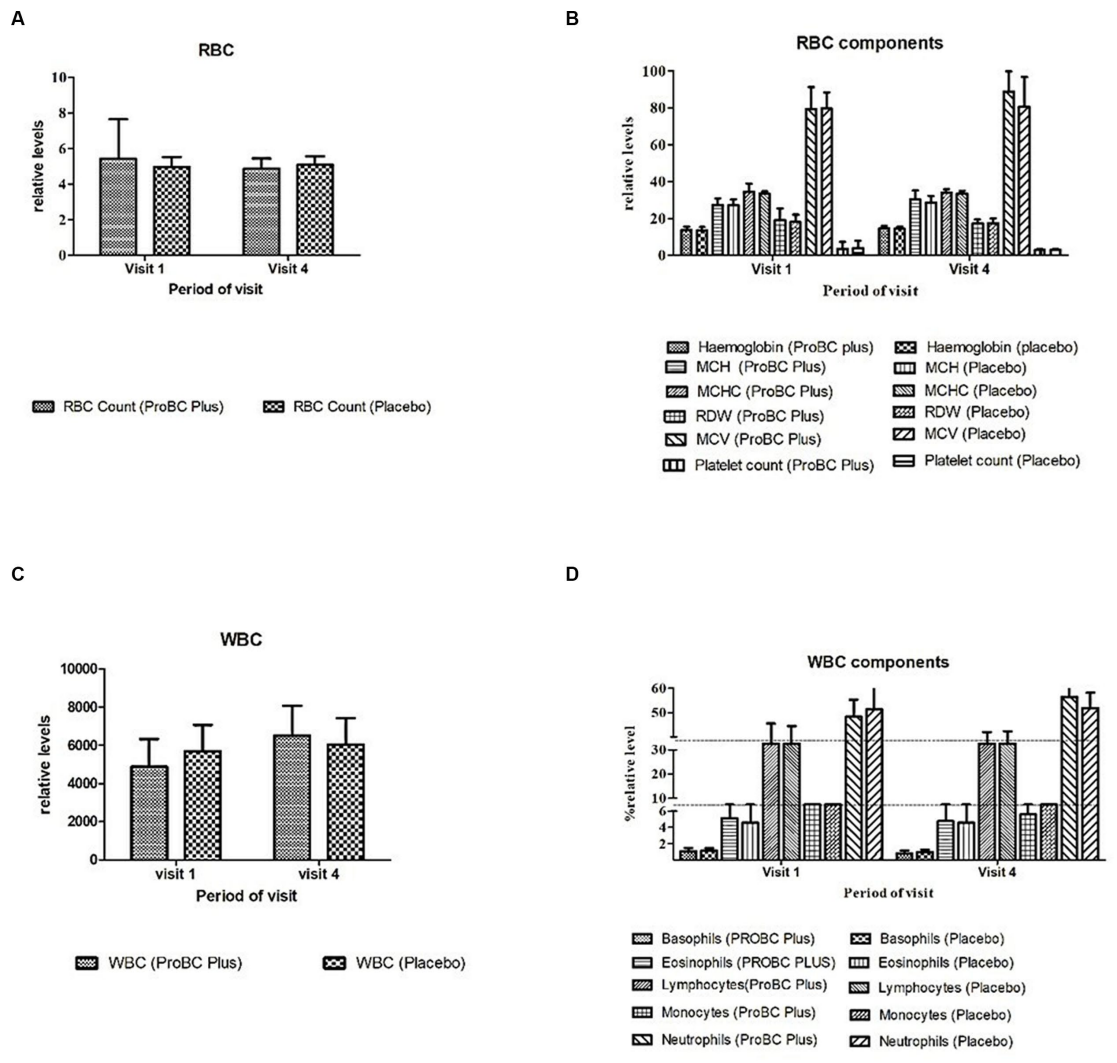
Parameters of hematology [RBC, its components, and WBC, its component **(A–D)**].

The hematology parameters indicated in Figure 3 depict a statistical significance concerning the baseline and revealed no statistical difference between the placebo and test product, and the ranges were within the reference levels.

#### 3.2.2 Estimation of lipid profiling

**Figure 4:**
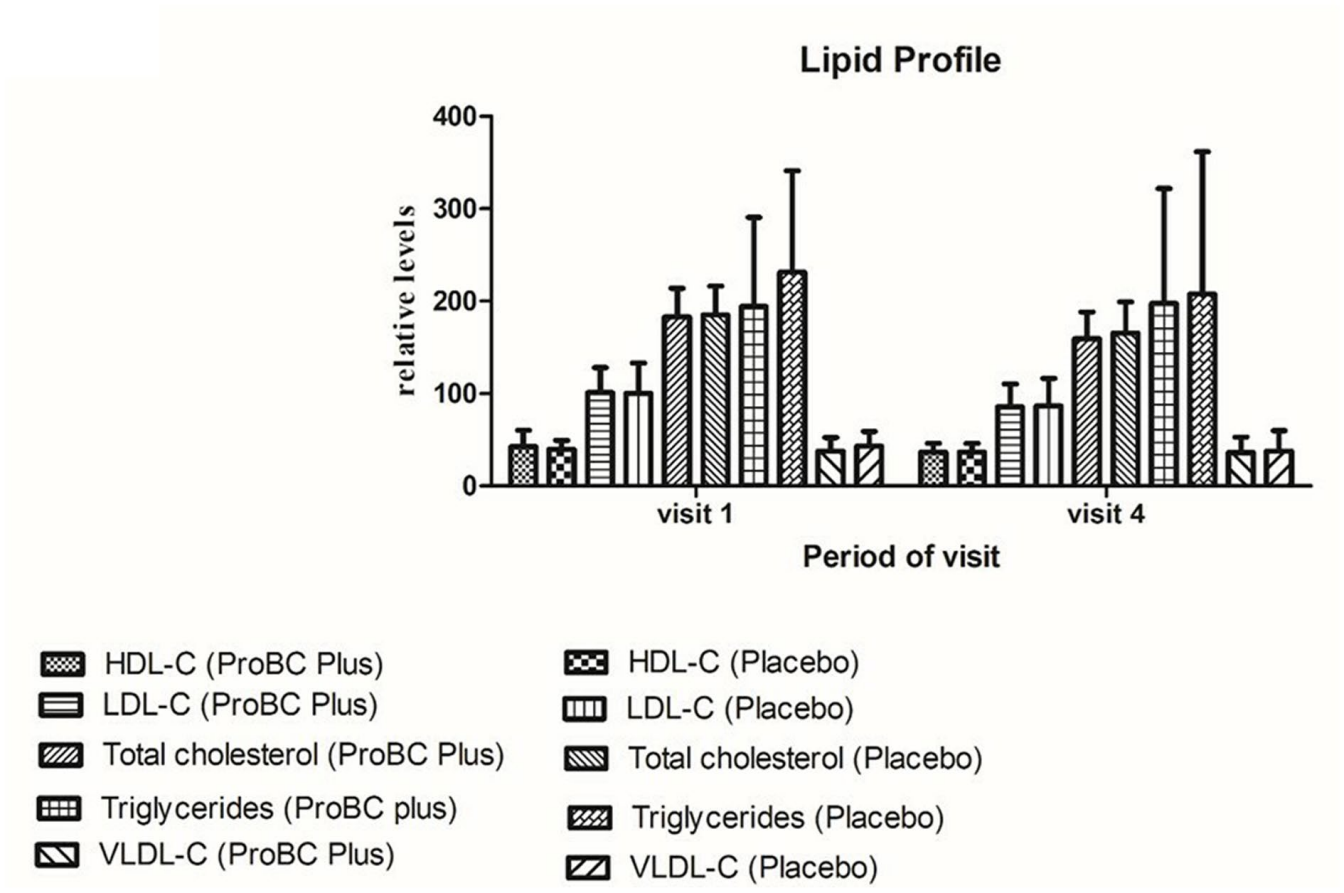
Analysis of lipid parameters (HDL—high-density lipoprotein; LDL—low-density lipoprotein; total cholesterol and triglycerides; VLDL—very low-density lipoproteins).

The lipid profiling parameters were found to be in the normal range (Figure 4). HDL (high-density lipoproteins), LDL (low-density lipoproteins), total cholesterol and triglycerides, and VLDL (very low-density lipoproteins) were found to be non-significant as compared to the placebo.

### 3.3 Analyzing the efficacy of the product

The common symptoms of IBS involve bloating, vomiting, and diarrhea. Changes in these symptoms were noticed throughout the visit.

VAS (Visual Analogue Scale), a value of 0 indicates “no pain,” while 10 represents “extreme pain.” A significant decline (*p* < 0.0001) from visit 1 to visit 4 depicts the efficacy of the test product as compared to the placebo. The change from baseline during visits 3 and 4 was estimated to be from −0.80 to −2.16 depicting the efficacy of the ProBC plus as compared to the placebo.

**Figure 5:**
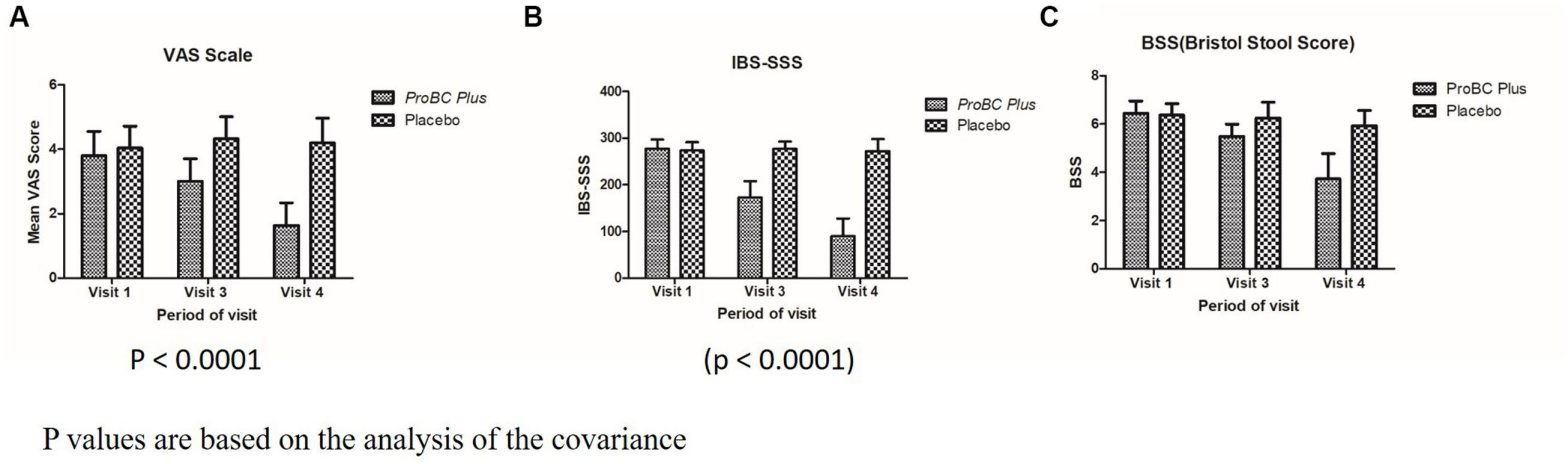
Analysis of covariance (*p*-value) **(A)** Mean VAS score with respect to period of visit **(B)** Mean IBS-SSS with respect to period of visit **(C)** BSS with respect to period of visit.

The other parameter, i.e., IBS-SSS (IBS Symptom Severity Scale) is a score that evaluates the abdominal pain/bloating (Figure 5). Supplementary Table S2 represents the significant change (decline) in the score as compared to the placebo (*p* < 0.0001). The change in the baseline from weeks 3 to 5 in the case of the test product ranged from −15.2 to −68.2 representing the efficacy of the test product as compared to the placebo.

In case of BSS (Bristol Stool Score) that is analyzed for stool frequency, the score reduction from 6.44 to 3.72 from visit 1 to visit 4 and the variation in baseline values from −0.96 to −2.72 depict the efficacy of the treatment with ProBC Plus as compared to the placebo.

## 4 Discussion

The current study was carried out to investigate the effects of ProBC Plus in study participants treated for IBS and its related symptoms. Imbalances in the microbiota of the gastrointestinal tract could exert significant alterations. IBS symptoms such as bloating, diarrhea, vomiting, and abdominal pain could be one of the major symptoms of gastrointestinal discomfort (22, 23).

Probiotics are very often defined as live microorganisms which when administered in optimum quantity possess a health benefit to humans which attracted a lot of attention during subsequent years (24–27). There have been quite a few studies that involve the utilization of probiotics and the delivery of probiotics in the form of capsules (28). Kank et al. (29) reported usage of *Weizmannia (Bacillus) coagulans as a dietary* supplement has significantly improved bowel movements (29). There is a continuous increase in demand for functional foods containing probiotic strains due to the awareness of the benefits for gut health and disease prevention and therapy, which also includes the potential of these strains to treat various forms of gastroenteritis (30, 31). However, health benefits provided by probiotics are strain specific, no probiotic strain will provide all proposed benefits, not even strains of the same species, and not all strains of the same species will be effective against defined health conditions (32–35).

In the present study, the study participants were divided equally into test and placebo. The parameters such as vital signs, hematology, and biochemical exhibited a normal range, and there were no significant differences between the baseline to the last visit, i.e., visit 8, and also placebo. The other important parameters such as VAS (Visual Analogue Scale) depicted a significant result as compared to the placebo; subsequently, BSS (Bristol Stool Scale) and SSS (Symptom Severity Scale) determined the efficacy of the product as compared to the placebo. This important observation establishes that the product ProBC Plus is safe to use and has no adverse events (AEs) or serious adverse events (SAEs). A similar type of result was obtained from the *Weizmannia (Bacillus) coagulans* LBSC and MTCC 5856 (22). Vaclav and Jana (36) reported that the probiotic *Bacillus coagulans* MTCC 5856 in combination with an aqueous extract of cinnamon has strong synergetic effects on phagocytosis and regulation of cholesterol and blood sugar levels and also confirmed that the combination reduced intestinal damage in a mouse model of colitis (37). There are quite a few studies indicating the utilization of *Weizmannia (Bacillus) coagulans* in reducing the symptoms of irregular bowel movements; according to one of the studies, the strain GBI-30, 6,086 is reported for the decline in baseline intensity score for 8 weeks as compared to the placebo (36). Similarly, in the present study, ProBC plus was found to be significant as compared to the placebo, and also, the baseline intensity score was reduced in the case of the VAS scale; in the treatment group, the baseline range was approximately 1.36, whereas, in the placebo group, it was approximately 0.12; similarly in IBS-SSS, the baseline range in the treatment group was approximately 52.8 and in the placebo was approximately 5.0 depicting high significant results. With BSS, in the treatment group, the baseline range was approximately 1.76, whereas in the placebo was approximately 0.32, which signifies the efficacy of ProBC Plus concerning the placebo.

Furthermore, it was also noticed that the mechanistic approach of *Weizmannia (Bacillus) coagulans* LMG S-31876 in the treatment of IBS-related issues can be a beneficial effect as it helps in suppressing the binding and further growth of pathogenic bacteria and improving the functions of the barrier and tight junctions (38). However, the possible mechanism could be suppression of the growth and binding of pathogenic bacteria, improvement in the barrier function of the epithelium, and alteration of the immune activity of the host as reported by Majeed et al. (39) wherein animal studies with *B. coagulans* MTCC 5856 elicited anti-diarrhoeal activity and inhibited the gastrointestinal motility in fasted Rats (39–41). Also, these types of probiotics are known to produce the short-chain fatty acids, an action that results in decreased luminal pH and production of bactericidal proteins (42–45).

Furthermore, according to Skrzydło-Radomańska et al. (46), in a prospective double-blind placebo-controlled clinical trial, *Bacillus coagulans* BC 300 NORDBIOTIC signifies that the IBS-SSS score significantly decreased in the study group. Madempudi et al. (47) exhibited that *Bacillus coagulans* Unique IS2 was efficacious in lowering abdominal pain, bloating, and stool consistency in IBS patients. Similarly, *Bacillus coagulans* LBS has improved the IBS symptoms that include abdominal pain in the intervention group as compared to the placebo (48). ProBC Plus had a similar impact of reducing symptoms such as abdominal pain, bloating, diarrhea, and stool frequency and had marked a remarkable difference as compared to the placebo.

## 5 Conclusion

The present study depicts a substantial reduction in clinical symptoms associated with irritable bowel syndrome and diarrhea (IBS-D). In particular, symptoms such as abdominal pain, bloating, diarrhea, and stool frequency depicted a significant decline and exhibited a marked improvement as compared to the placebo. Furthermore, the comprehensive analysis of the parameters such as hematology, vital signs, and lipid profile were found to be in the normal range with the supplementation of ProBC Plus throughout the period of 8 weeks. Therefore, this steadfastly reinforces the safety of the probiotic product ProBC Plus. Subsequently, the results indicate the notion that *Weizmannia (Bacillus) coagulans* LMG S-31876, administered in the form of ProBC Plus at a dosage of 2 billion CFU/day, exhibits therapeutic influence in managing clinical symptoms associated with IBS-D prominently and provides a great promise in the enhancement of the quality of the life for the individual grappling with the IBS-D issues.

### 5.1 Recommendation

Individuals experiencing symptoms of irritable bowel syndrome and diarrhea (IBS-D) consider the incorporation of ProBC Plus, containing *Weizmannia* (*Bacillus*) *coagulans* LMG S-31876 at a dosage of 2 billion CFU/day, as a potential therapeutic intervention.

## Data availability statement

The datasets presented in this study can be found in online repositories. The names of the repository/repositories and accession number(s) can be found in the article/Supplementary material.

## Ethics statement

The studies involving humans were approved by Medstar Specialty Hospital, Bengaluru. The studies were conducted in accordance with the local legislation and institutional requirements. The participants provided their written informed consent to participate in this study.

## Author contributions

RK: Conceptualization, Formal Analysis, Supervision, Writing – original draft. SM: Project administration, Resources, Supervision, Writing – review & editing. AM: Data curation, Formal Analysis, Investigation, Methodology, Validation, Writing – original draft. SB: Writing – review & editing.
