# Supplementary material for: The role of Weizmannia (Bacillus) coagulans LMG S-31876 in treating IBS-diarrhea
Source: Front Nutr. 2024 Feb 5;10:1310462. doi: 10.3389/fnut.2023.1310462 (PMC10875997; doi:10.3389/fnut.2023.1310462)
Supplement: Supplementary file 1 [file Table_1.DOCX]

**Table S1: Summary of Demographic Characteristics ITT Population**

Table S1
Summary of Demographic Characteristics ITT Population

|  | Statistics | Study Drug (N=25) | Placebo (N=25) |
| --- | --- | --- | --- |
| Age (Yrs) |  |  |  |
|  | n | 25 | 25 |
|  | Mean | 34.20 | 40.68 |
|  | SD | 10.10 | 10.21 |
|  | Median | 33.00 | 40.00 |
|  | Min, Max | 18,50.00 | 21,60.00 |
|  |  |  |  |
| Weight (Kg) |  |  |  |
|  | n | 25 | 25 |
|  | Mean | 62.36 | 68.04 |
|  | SD | 9.07 | 8.11 |
|  | Median | 61.00 | 70.00 |
|  | Min, Max | 49,79.00 | 54,82.00 |
|  |  |  |  |
| Height (cm) |  |  |  |
|  | n | 25 | 25 |
|  | Mean | 160.9 | 163.1 |
|  | SD | 7.30 | 7.18 |
|  | Median | 159.00 | 163.00 |
|  | Min, Max | 150,180.00 | 150,176.00 |
|  |  |  |  |
| Gender |  |  |  |
|  |  |  |  |
| Female | n (%) | 14 (56.0) | 9 (36.0) |
| Male | n (%) | 11 (44.0) | 16 (64.0) |
|  |  |  |  |
| Race |  |  |  |
| Asian | n (%) | 25 (100.0) | 25 (100.0) |
