# Supplementary material for: The role of Weizmannia (Bacillus) coagulans LMG S-31876 in treating IBS-diarrhea
Source: Front Nutr. 2024 Feb 5;10:1310462. doi: 10.3389/fnut.2023.1310462 (PMC10875997; doi:10.3389/fnut.2023.1310462)
Supplement: Supplementary file 2 [file Table_2.DOCX]

**Table S2: Categorical Analysis of Bristol Stool Form Scale (BSS)**

**ITT Population**

| Visit | Bristol stool form scale | Study Drug  (N=25)  n (%) | Placebo  (N=25)  n (%) |
| --- | --- | --- | --- |
|  |  |  |  |
| Visit 1 | 6 | 14 (56.0%) | 16 (64.0%) |
|  | 7 | 11 (44.0%) | 9 (36.0%) |
|  |  |  |  |
| Visit 3 | 5 | 13 (52.0%) | 3 (12.0%) |
|  | 6 | 12 (48.0%) | 13 (52.0%) |
|  | 7 | 0 | 9 (36.0%) |
|  |  |  |  |
| Visit 4 | 2 | 4 (16.0%) | 0 |
|  | 3 | 6 (24.0%) | 0 |
|  | 4 | 8 (32.0%) | 0 |
|  | 5 | 7 (28.0%) | 6 (24.0%) |
|  | 6 | 0 | 15 (60.0%) |
|  | 7 | 0 | 4 (16.0%) |
| ________________________________________________________________________ | | | |
